# Long‐lived larch clones may conserve adaptations that could restrict treeline migration in northern Siberia

**DOI:** 10.1002/ece3.6660

**Published:** 2020-08-17

**Authors:** Stefan Kruse, Aleksey I. Kolmogorov, Luidmila A. Pestryakova, Ulrike Herzschuh

**Affiliations:** ^1^ Polar Terrestrial Environmental Systems Alfred Wegener Institute, Helmholtz Centre for Polar and Marine Research Potsdam Germany; ^2^ Institute of Natural Sciences North‐Eastern Federal University of Yakutsk Yakutsk Russia; ^3^ Institute of Environmental Sciences and Geography University of Potsdam Potsdam Germany; ^4^ Institute of Biology and Biochemistry University of Potsdam Potsdam Germany

**Keywords:** adaptation, clonal growth, growth rate, *Larix*, leading edge, treeline migration

## Abstract

The occurrence of refugia beyond the arctic treeline and genetic adaptation therein play a crucial role of largely unknown effect size. While refugia have potential for rapidly colonizing the tundra under global warming, the taxa may be maladapted to the new environmental conditions. Understanding the genetic composition and age of refugia is thus crucial for predicting any migration response.

Here, we genotype 194 larch individuals from an ~1.8 km^2^ area in northcentral Siberia on the southern Taimyr Peninsula by applying an assay of 16 nuclear microsatellite markers. For estimating the age of clonal individuals, we counted tree rings at sections along branches to establish a lateral growth rate that was then combined with geographic distance.

Findings reveal that the predominant reproduction type is clonal (58.76%) by short distance spreading of ramets. One outlier of clones 1 km apart could have been dispersed by reindeer. In clonal groups and within individuals, we find that somatic mutations accumulate with geographic distance. Clonal groups of two or more individuals are observed. Clonal age estimates regularly suggest individuals as old as 2,200 years, which coincides with a major environmental change that forced a treeline retreat in the region.

We conclude that individuals with clonal growth mode were naturally selected as it lowers the likely risk of extinction under a harsh environment. We discuss this legacy from the past that might now be a maladaptation and hinder expansion under currently strongly increasing temperatures.

## Graphical abstract

The study features findings of clonal growth and potentially millennial‐long survival in refugia beyond the treeline in northern Siberia for the first time. We conclude that surviving individuals were naturally selected for the ability to spread clonally as it lowers the likely risk of extinction under a harsh environment. We discuss this legacy from the past that might now be a maladaptation and could restrict treeline migration under currently strongly increasing temperatures.

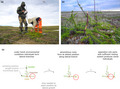

## 1 INTRODUCTION

Climate warming causes environmental changes, and plant species are expected to expand their distribution at the leading edge. However, for tree species the timing and rate depends mainly on dispersal potential and survival of the offspring under changed conditions (Clark et al., 1998; Hampe, 2011). This is constrained by population genetics, and genetically diverse populations are expected to perform better generally under a broad range of environments. The genetic diversity of a population can be shaped differently when climate changes: It can either avoid unfavorable environmental changes by contracting its distribution area, or genetically adapt to its environment via sexual reproduction that enables the mixing of parental genotypes (crossover) and subsequent selection of the produced genotypes (Arenas, Ray, Currat, & Excoffier, 2012). This latter process needs multiple generations to occur, so if the environmental change is too fast and the population fails to adapt it can go extinct. Alternatively, individuals may survive in refugia, but typically the small population size and increased genetic drift increase niche adaptation, which may be a disadvantage by hindering responses should the environment improve again (Ackerly, 2003; Arenas et al., 2012; e.g., niche adaptation, genetic drift, Pearman, Guisan, Broennimann, & Randin, 2008). Understanding the genetic population history is the key to deducing realized migration under a currently changing climate.

With the current strong warming rate, the hypothetical suitable area for forest extends northwards by about 1 km/yr (Loarie et al., 2009). Therefore, the taiga could already potentially replace tundra for several kilometers along the approximately 5,000 km long treeline (Epstein et al., 2004; but see Harsch, Hulme, McGlone, & Duncan, 2009; MacDonald, Kremenetski, & Beilman, 2008; Mamet, Brown, Trant, & Laroque, 2019). The colonization of tundra by trees has a net warming effect and will thus cause a positive feedback to globally warming temperatures (Bonan, 2008), yet the rate of change is largely unknown because the processes involved in treeline migration are not that well understood (MacDonald et al., 2008; Mamet et al., 2019). Modern migration rates at the latitudinal treeline in Siberia are only a few meters per year (Kharuk, Ranson, Im, & Naurzbaev, 2006; Kruse et al., 2019). While restriction of forest expansion arising from seed dispersal ability is comparatively well studied (Harsch et al., 2009; Holtmeier & Broll, 2020; Wieczorek et al., 2017), the impacts of treeline form and its gene pool are not well studied.

The different treeline forms can be used to assess whether a treeline is able to respond rapidly to improvements in the environment or can only respond linearly, which, in general, is slow (Harsch et al., 2009). The form generally mirrors the environment and can be either abrupt (steep climate gradient, usually elevational) with some trees surviving at favorable microsites (refugia) beyond the treeline or stretched over transition zones (long gradient, usually latitudinal; Holtmeier & Broll, 2005). At many treeline areas, krummholz occurs, formed of layered/stunted individuals growing close to the ground because conditions are unfavorable for upright growth forms (cf. Cooper, 1911; Del Tredici & Orwig, 2017). This krummholz might promote tundra invasion when conditions improve (Devi et al., 2008; Holtmeier & Broll, 2005; Kharuk et al., 2006; Martínez, Wiegand, Camarero, Batllori, & Gutiérrez, 2011). Further, the lateral branches can produce adventitious roots when touching the ground, which is a widespread phenomenon reported especially for species of the Pinaceae family growing at the treeline in high elevations (Cooper, 1911). When the connection rots, genetically identical individuals could be found. This ability to reproduce asexually by layering has ecological significance as it can increase the survival rate in the short term at least: first by spreading the risk of extinction of a successful genotype by having several genetically similar individuals in the area, and second by having temporarily connected ramets that can support each other (found e.g., for *Tsuga canadensis* Del Tredici & Orwig, 2017). It is argued that this reproduction mode would allow populations to survive adverse times, but also it enables the transmission of genetic information through time (Arnaud‐Haond et al., 2006; Balloux, Lehmann, & de Meeûs, 2003), which comes with a threat: Individuals may find themselves maladapted to new situations. Survival in refugia may come with huge costs: The taxa could carry historical adaptations to colder environments making them maladapted to the current strong warming and thereby restricting tundra colonization (Aitken, Yeaman, Holliday, Wang, & Curtis‐McLane, 2008; Johnson, Chhetri, Krutovsky, & Cairns, 2017). Unfortunately, the roles of genetic adaptation and clonal reproduction of krummholz, which potentially enables millennia‐long survival of single genotypes in the landscape (Laberge, Payette, & Bousquet, 2000), in migration dynamics are not yet understood.

The responses at different locations around the world are diverse (Harsch et al., 2009) and especially unclear at the Siberian latitudinal treeline. In the past, the treeline dynamically changed position under varying climates over millennia (MacDonald et al., 2008). However, on a decadal–centennial timescale, a pronounced time lag is caused by the species' long life cycles and their poor dispersal ability so that Siberian treelines are in disequilibrium with the current warming and the expected strong response to global warming only started recently (Kruse et al., 2019; Kruse, Wieczorek, Jeltsch, & Herzschuh, 2016; Mamet et al., 2019; Wieczorek et al., 2017). It is unknown whether the historical genotypes are responsible for the unexpectedly slow responses to global warming, as observed at the Siberian treeline, or how they will shape future migration dynamics. Furthermore, with a subsequent influx of other genotypes (by pollen or seed), hybrids may form (Aitken et al., 2008; effect called gene swamping, e.g., Johnson et al., 2017) bringing another unknown dynamic into play.

Krummholz refugia in northern Siberia are thought to have survived for thousands of years. The age of refugia has been estimated for other krummholz‐forming Pinaceae species, and ages of up to 1,800 years were inferred (clonal groups of Black Spruce Laberge et al., 2000) from remains of individuals below the canopy. This dating approach is appropriate for stationary individuals, but larch (*Larix* Mill.) can grow adventitious roots from their branches if they touch the ground (Cooper, 1911; Kajimoto, 2010). Consequently, clonal groups can be observed (Holtmeier & Broll, 2017) and, if the clones then become separated or the initial individual from which the krummholz group has formed dies, separate individuals sharing the same genotype will occur. Without the remains of former parts of an individual, the Laberge et al. (2000) dating approach is not applicable. Instead, we assume that the age of such clonal individuals can be estimated by assessing their lateral growth rate, and we expect that the direction might be traceable genetically because somatic mutations can occur during this process which would be observable via the accumulated differences in genotypes by distance (e.g., Gross, Nelson, Haddadchi, & Fatemi, 2012; O'Connell & Ritland, 2004, but see also Cloutier, Rioux, Beaulieu, & Schoen, 2003).

Present‐day individuals need to be genotyped to trace back their genealogy and to understand the reproduction mode in krummholz refugia. This can be accomplished by regularly applying neutral genetic markers specifically designed for larches in Siberia (Isoda & Watanabe, 2006; Oreshkova, Vetrova, & Sinelnikova, 2015; Wagner, Gerber, & Petit, 2012) to assess the state of the population dynamics (Ashley, 2010; Selkoe & Toonen, 2006). The revealed genealogy for a population can help distinguish between sources of recruitment (local vs. external) and the reproduction mode (sexual vs. clonal). However, as each individual from a population must be genotyped to reconstruct the full genealogical network, this method is of limited use when population sizes are too large to genotype all potential parents and relatives (Ashley, 2010; Selkoe & Toonen, 2006). The small population size of single tree stands beyond the treeline in northern Siberia (Wieczorek et al., 2017), however, is suitable for this method and their distribution means large spatial areas can be covered.

This study aims to help understand the role of krummholz in treeline dynamics. First, we distinguish the origin of selected individuals as either via sexual reproduction (by pollination and seed dispersal) or asexual clonal growth by genotyping with highly polymorphic nuclear microsatellite markers. Second, this information combined with the lateral growth rate and distances between individuals of clonal origin allows us to estimate since when genotypes have survived in the refugia. Based on the evidence, we discuss the consequences of historical genotypes for the current and future treeline in response to a changing climate.

## 2 MATERIAL AND METHODS

### 2.1 Field site description and sampling protocol

**FIGURE 1:**
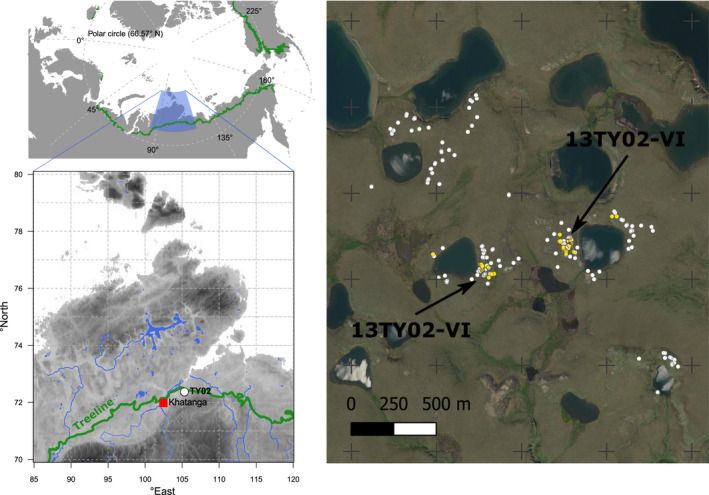
Overview of the study region located in the treeline margin of boreal forests close to the settlement of Khatanga on the southern Taimyr Peninsula (left). Present larch individuals were exhaustively sampled (one sample per white dot, >1 sample per yellow dot) in a heterogeneous permafrost landscape of prostrate tundra with many thermokarst lakes (right). The green line marks the modern maximum position of the treeline (Walker et al., 2005). Elevation indicated by gray shading (WorldClim1.4, Hijmans, Cameron, Parra, Jones, & Jarvis, 2005). Rivers and lakes are shown in blue colors (GSSHS updated version 2.2.2 01.01.2s013 first published by Wessel & Smith, 1996). The background image © Esri World Imagery

**FIGURE 2:**
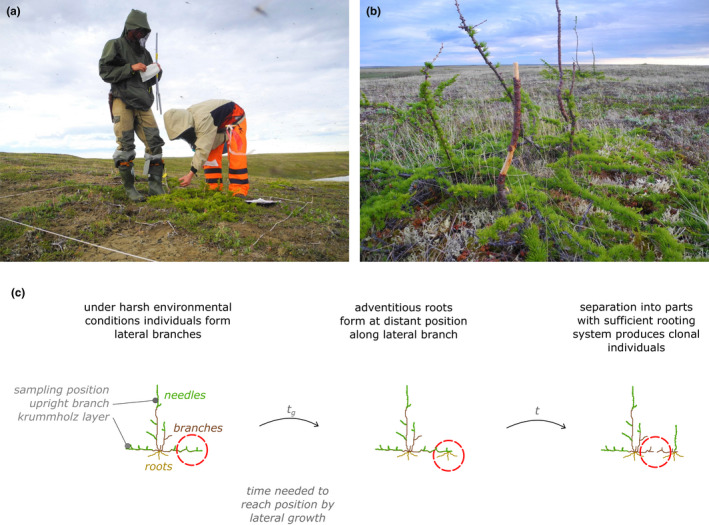
Examples of krummholz individual analysis: (a) Aleksey Kolmogorov and Bastian Niemeyer investigating a very low creeping krummholz individual; (b) close‐up of a krummholz individual with damaged upright stems. (c) Schematic diagram of clonal reproduction by lateral growth/layering. Images by Stefan Kruse

During the Russian–German summer expedition “Taimyr 2013” to the lowlands on the southern Taimyr Peninsula, we visited a tundra region with a very low density of the deciduous boreal tree species *Larix gmelinii* (Rupr.) Rupr. growing at favorable sites (TY02, 72.549°N, 105.745°E, https://doi.org/10.1594/PANGAEA.870947; Figure 1). This area is located ~15 km beyond continuous forest cover and the latitudinal treeline in northcentral Siberia. The observed, dominant growth form of larches is layered close to the surface with occasional upright stems that are often dead or damaged, forming so called krummholz (Figure 2a). We defined a group of branches originating from the same stem base as one individual when no aboveground connection to other such groups was visible (Figure 2c). For close growing individuals, we checked for any belowground connection by following the roots, but found no linking roots in any instance.

Following our sampling scheme for genetic analyses, a total of 227 needle samples from 194 individuals were taken from across an area of ~1.8 km^2^ (Figure 1, right). The sampling consisted of three steps with increasing resolution. First, to retrieve the full genetic structure of the population in the visited region TY02, we took at least one sample from all individuals spotted during the visit. For each individual, position was recorded using a handheld GPS along with height and crown cover measurements. Second, within this region, two sites TY02‐VI and TY02‐VII were chosen for a detailed forest structure and vegetation analysis, which was the aim of the expedition (Kruse et al., 2018). At these sites—characterized by relatively dense krummholz growth—in addition to recording the position and properties of each individual, we took needles from the tallest upright branch of each individual and, in 20 cases, sampled at least once the layered krummholz most distant from the first sample at ground‐level. Finally, from three individuals (L060, L039, L139), we sampled all the living lateral branches and cut them into 10‐cm sections, which were brought to Germany for age determination. All information about sampled individuals is stored in PANGAEA (https://doi.org/10.1594/PANGAEA.920680).

### 2.2 Genotyping of individual larch samples

For Gmelin larch (*Larix gmelinii* (Rupr.) Rupr.) growing on the southern Taimyr Peninsula, a set of 16 markers have been selected, multiplexed in three groups, and applied to infer the population dynamics at the treeline ecotone (Table 1; details in Kruse et al., 2016).

**Table 1:** Summary table for the 16 nuclear microsatellite loci applied to all 194 individual samples from the region TY02 and at two sites TY02‐VI and TY02‐VII within the region, in northcentral Siberia

Locus^1^	TAG^2^	Repeat motif^3^	Size (bp)	*N*_A_	*H*_O_	*H*_E_	HWE	Evenness	Missing alleles	*F*_ST_	*F*_IS_
Multiplex 1											
bcLK241	Q1	(AG)_12_	157–201	19	0.36	0.91	*	0.77	12.89%	0.025	0.599
bcLK066	Q3	(TG)_12_	154–172	9	0.53	0.74	*	0.71	–	0.005	0.291
bcLK253	Q3	(AG)_17_	217–247	16	0.85	0.81	*	0.62	–	0.019	−0.035
bcLK211	Q2	(CT)_16_	196–240	22	0.86	0.91	*	0.74	–	0.022	0.052
Ld101	Q4	(AC)_12_	206–236	15	0.60	0.78	*	0.58	–	0.054	0.206
Multiplex 2											
bcLK260	Q1	(TG)_14_(AG)_9_	103–127	12	0.28	0.84	*	0.83	–	0.056	0.654
bcLK056	Q1	(AG)_20_	164–232	26	0.67	0.93	*	0.78	2.06%	0.015	0.276
bcLK224	Q3	(AG)_17_	140–158	8	0.30	0.68	*	0.70	8.25%	0.047	0.545
Ld45	Q3	(CA)_13_	214–240	9	0.31	0.71	*	0.63	8.76%	0.015	0.566
bcLK263	Q2	(TC)_20_	198–274	36	0.92	0.94	*	0.71	–	0.012	0.014
bcLK228	Q4	(AG)_18_	187–229	18	0.78	0.90	*	0.83	–	0.017	0.123
Multiplex 3											
bcLK225	Q1	(GA)_20_	171–225	24	0.47	0.90	*	0.68	11.86%	0.021	0.474
bcLK235	Q3	(TC)_9_(AC)_2_AG(AC)_14_	137–245	23	0.71	0.89	*	0.67	3.61%	0.009	0.204
bcLK189	Q2	(AG)_17_AT(AG)_6_	152–230	28	0.85	0.90	*	0.67	1.03%	0.012	0.044
Ld56	Q2	(AC)_16_	247–275	15	0.28	0.81	*	0.63	9.28%	0.061	0.644
Ld42	Q4	(TG)_14_	187–201	8	0.62	0.79	*	0.82	0.52%	0.029	0.200
Mean				18	0.59	0.84		0.71	3.64%	0.026	0.304

Note

For each locus, the observed size range and the number of different alleles (*N*_A_) are given, and observed and expected heterozygosity by *H*_O_ and *H*_E_; significant deviations from Hardy–Weinberg equilibrium (HWE) with *p* < .001 are denoted with *; missing alleles give the fraction of individuals with no amplified alleles at the loci if any; *F*‐statistics are calculated using the three groups TY02, TY02‐VI, and TY02‐VII.

^1^ Marker names beginning “bcLK” are developed by Isoda and Watanabe (2006) and those with “Ld” by Wagner et al. (2012); Multiplex—number indicates the three primer mixes applied in a simultaneous PCR.

^2^ Tailing sequence at forward primer: Q1 = TGTAAAACGACGGCCAGT (Schuelke, 2000); Q2 = TAGGAGTGCAGCAAGCAT; Q3 = CACTGCTTAGAGCGATGC; Q4 = CTAGTTATTGCTCAGCGGT (Q2–Q4, after (Culley, Weller, Sakai, & Putnam, 2008)); fluorescent‐labeled reverse primers for each tag were Q1 = 6‐FAM™, Q2 = NED™, Q3 = VIC®, and Q4 = PET™.

^3^ Motif information from original publication as specified in this table footnote 1.

The genotyping steps, (a) DNA extraction, (b) multiplexed PCR, and (c) microsatellite fragment length determination, follow our laboratory protocol established and applied earlier (Kruse et al., 2018, 2019). The DNA was extracted from approximately 100 mg dried needle sample material with the Invisorb® Spin Plant Mini Kit (STRATEC MOLECULAR) or DNeasy Plant Mini Kit (QIAGEN), following the supplier's protocol, with modifications.

PCRs of 10 μl were set up using the Multiplex PCR Master Kit (QIAGEN), containing ~1–5 ng template DNA, primer (0.7 μM forward, 1.0 μM reverse, and 1.0 μM fluorescent‐labeled reverse primer for Q1‐Q4 tags) and MasterMix (containing 25 units HotStarTaq® DNA polymerase, Multiplex PCR Buffer (pH 8.7) with a final 3 mM MgCl_2_ and dNTP Mix).

PCRs were performed using the following reaction profile: 15‐min activation of polymerase and initial DNA melting at 94°C, followed by 20 cycles of 30 s 94°C, 90 s 60°C, and 15 s 72°C, a further 10 repeats for 30 s 94°C, 45 s 53°C, and 15 s 72°C and a final elongation step at 60°C for 45 min to minimize stutter of bands introduced by incomplete A‐tailing of products (QIAGEN manual for Multiplex PCR Master Kit).

Fragment length estimation was performed by SourceBiosciences (Oxford, UK), with 1 μl of each PCR product added to 9 μl HiDi formamide/size standard mixture LIZ 600®, dye set DS‐33, and run on an ABI 3730xl DNA Analyser. The resulting raw data were processed in Geneious (version 7.1.5, Biomatters Ltd.) using the Microsatellite plug‐in (version 1.4.0, Biomatters Ltd.), and the first automatically scored peaks were checked and adjusted manually. Subsequent binning of scored alleles was calculated in R using a locally weighted method and previously provided scripts (Kruse, 2018). Fragment length data for all processed samples are stored in PANGAEA (https://doi.org/10.1594/PANGAEA.920680).

For samples of the same individual that showed differently binned alleles (see Appendix S5 for examples), we ran the PCR and fragment length analyses a second time to validate the results (similar to the methodology of O'Connell & Ritland, 2004). For all of them, the same allele lengths were observed in the second run, and thus, we handle allele differences as somatic mutations (not shown).

### 2.3 Data processing and analyzing allelic data

We used R version 3.6.1 (R Core Team, 2019) for postprocessing, analyzing, and plotting the data. To transform the allele information into a genind object for subsequent analyses, we used the R package “adegenet” version 2.1.1 (Jombart, 2008; Jombart & Ahmed, 2011).

For a summary of the scored alleles per loci, a subset of the 194 separate individuals was used. For identifying clonal connections, we calculated the absolute allele differences between all 16 loci (“diss.dist” function from “poppr” version 2.8.3 (Kamvar, Brooks, & Grünwald, 2015; Kamvar, Tabima, & Grünwald, 2014)). Connections with no allelic differences were counted as clonal and those with a maximum of up to 3 observed different alleles as clonal with somatic mutations. Further, we made a subset of genotypes that consist only of one remaining genotype per clone group. The number of individuals originating from clonal growth was calculated by subtracting those genotypes that occur only once in the region and the number of clonal groups from the total number of individuals in the region. Dividing this by the total number of individuals yields a clonal growth excess rate.

The number of loci necessary to differentiate between all individual genotypes (multilocus genotypes) was determined with the “genotype_curve”‐function from R package “poppr” by permuting loci for 100 times and counting the number of multilocus genotypes observed.

For the clone and somatic mutation‐censored data set, we calculated the observed and expected heterozygosity and tested the departure from the Hardy–Weinberg equilibrium (HWE) with the “hw.test”‐function from the R package “pegas” version 0.13 (Paradis, 2010), which was used to make exact tests based on 1,000 Monte Carlo permutations of alleles. *F*‐statistics (*F*_ST_, *F*_IS_) are calculated after Weir and Cockerham (1984) via the “Fst”‐function from R package “pegas” assigning samples into three groups: the region TY02, and the sites TY02‐VI and TY02‐VII.

To differentiate between sexual and asexual/clonal reproduction within the population, we calculated the index of association (*I*_A_) using the “ia”‐function from R package “poppr.” Following Goss et al. (2014), clonal reproduction mode can be assumed when index values are significantly larger than the distribution calculated from a 1,000‐fold permuted data set of all three data sets: (a) including all individuals, (b) clone‐censored, and (c) clone and somatic mutation‐censored.

### 2.4 Age determination along branches for reconstructing lateral growth

Three separate groups (L060, L039, and L139) were selected in the field that had 2, 3, and 11 lateral branches. Each branch was cut into 10 cm‐long pieces, dried, and transported to our laboratory facilities in Potsdam for further analyses. From each branch, the oldest part and the middle piece were selected, but for the individual with the longest branch (branch H5 of L139, ~265 cm), we decided to analyze two further samples at distances half‐way between the oldest part and the middle and between the middle and the tip. Samples were sanded using sandpaper and decreasing the grain size until the surface was even and the tree rings clearly visible under a binocular microscope. All samples were scanned at 4,800 dpi on a flatbed scanner and tree rings recorded in the software Coorecorder (Cybis Elektronik & Data AB). The number of tree rings counted corresponds to the age of the respective section of the branch. The length along the branch between two age determinations was divided by the age difference to get the growth rate in cm/yr. All information is stored in PANGAEA (https://doi.org/10.1594/PANGAEA.920838).

The growth rate was then used to estimate the age of the present separate krummholz groups that share the same genotype or have accumulated one or a few allele differences. Distance between two samples was taken into account, and we selected the oldest estimated age in clonal groups with more than two individuals.

## 3 RESULTS

### 3.1 Genotyping of individuals

Across the whole data set of 194 visually separate individuals, 8–36 different alleles were found at the 16 applied microsatellites with 5 loci showing more than 5% missing alleles (Table 1). All loci deviate significantly from the Hardy–Weinberg equilibrium, and mean observed heterozygosity (0.59) is smaller than expected (0.84). Genotype evenness is high with values of 0.62–0.83 (mean 0.71).

**FIGURE 3:**
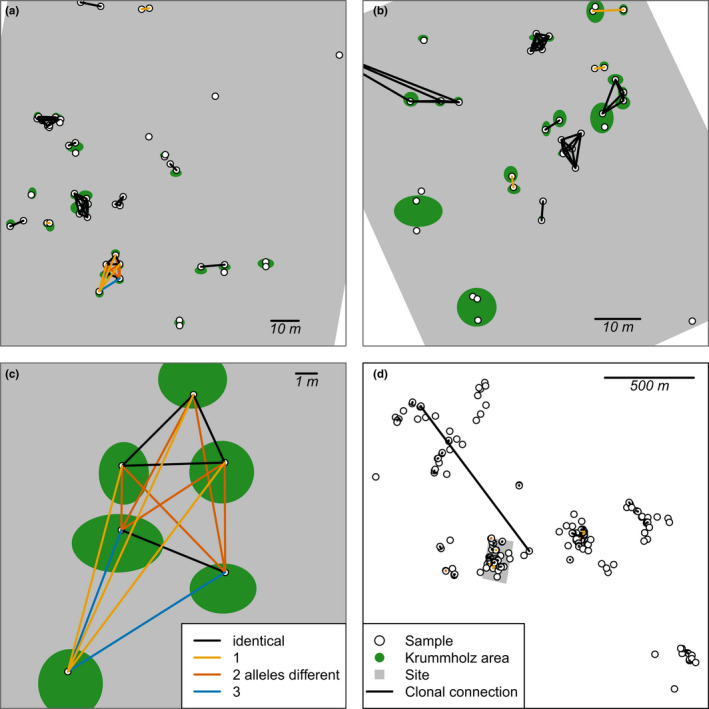
Many individuals share the same genotype or have 1–3 different alleles at the two sites (a: 13TY02‐VI, b: 13TY02‐VII). The within clonal‐group networks can be complex, and an example of a large group shows multiple levels of allele differences (c). One extreme distant clonal connection is clearly visible in the overview of all samples and clonal connections for the whole area (d)

**FIGURE 4:**
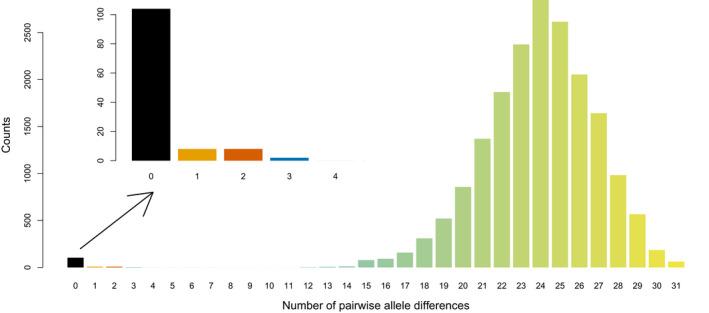
Distribution of pairwise genetic distance values across all analyzed individuals. The inset enlarges the clonal individuals with no genetic difference (black) or small genetic differences up to 3 different allele lengths (orange, dark orange, and dark blue). See further details in caption of Figure 5

**FIGURE 5:**
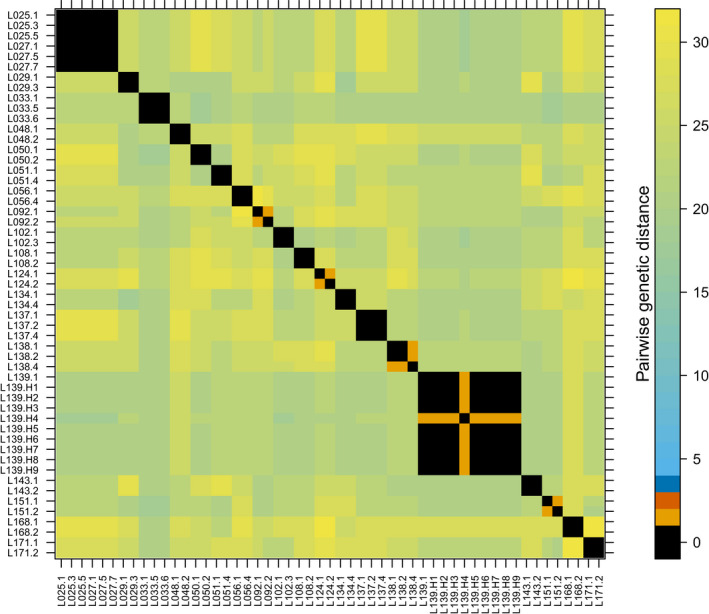
Genetic distance among individuals from which multiple samples were taken. Black colored clusters show genetically identical samples taken within the same individuals, and orange, dark orange, and dark blue show somatic mutations. Samples L025 and L027 (upper left corner) indicate genetically identical clusters across individuals. See further details in Appendix S4

The genotype accumulation curve showed that 5 out of 16 loci would be sufficient to distinguish between the multilocus genotypes when excluding clones and somatic mutations (Appendix S1). With the latter included, the 100% differentiation level is only approached when using 15 loci. We could differentiate 124 multilocus genotypes. Of these, 80 genotypes were only found once, whereas 44 formed clonal groups of two or more individuals (connected sample locations in Figure 3), leading to a clonal growth excess of 36.08%. We observed somatic mutations (1–3 allele differences) in seven groups of two individuals and one group with six individuals (see Figures 3c and 4, Appendix S3, Appendix S4). In addition, we observed somatic mutations in four individuals out of 20, revealed by differences at one microsatellite marker when comparing the within individuals' genotypes (Figure 5, Appendix S3).

The index of association (*I*_A_) was significant for the data set with all individuals (*p* < .001), became less significant in the clone‐censored subset (*p* = .0849), and insignificant for the clone and somatic mutation‐censored subset (*p* = .549; Appendix S2).

### 3.2 Age estimation within clonal groups

**FIGURE 6:**
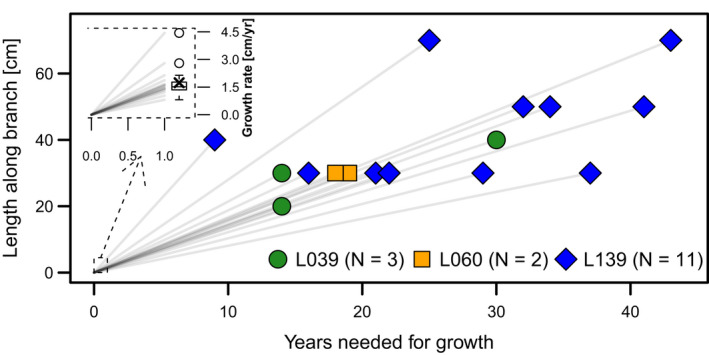
Lateral growth and ages along 16 branches of three analyzed krummholz individuals. The inset enlarges the growth in year one with the growth rate values inserted as a boxplot to the right, with the mean value indicated by an x

Assuming the distance between clonal individuals is equivalent with the time needed to reach the position by lateral growth, we can estimate the age of the individuals from the lateral growth rate and the geographic distance. The lateral growth rates are similar for the three analyzed individuals (Figure 6). The oldest branch segment is 44 years old with a length of 70 cm, and the youngest is 9 years but already 40 cm in length. The mean growth rate across the studied region is 1.74 ± 1.67 cm/yr.

**FIGURE 7:**
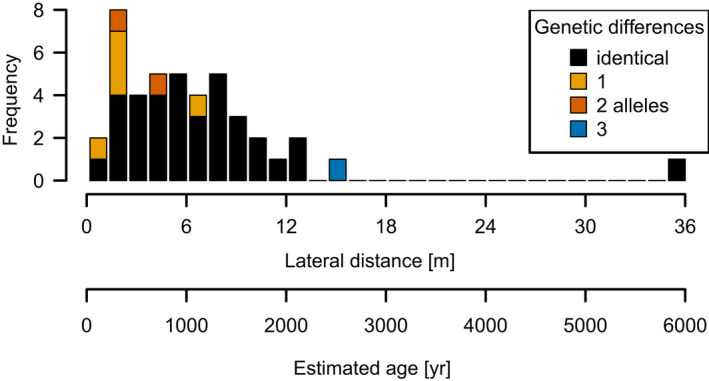
Geographic distance and estimated age for connections within clonal groups. Age data are aggregated in bins with widths of 200 years; one outlier at 1,015 m is not shown

The distribution of geographic distances between 122 genetically identical individuals is right‐skewed (median = 4.77 m, mean = 14.18 m). Excluding one outlier of 1,015 m, the largest distance is 35 m (Figure 7). From these distances, we estimated the age since separation by applying our estimate of the lateral growth rate. About 95% of the individuals are <2,200 years old (Figure 7). The longest connection was extrapolated to ~6,000 years and the outlier to 177,000 years.

### 3.3 Genetic differences across geographic distances

**FIGURE 8:**
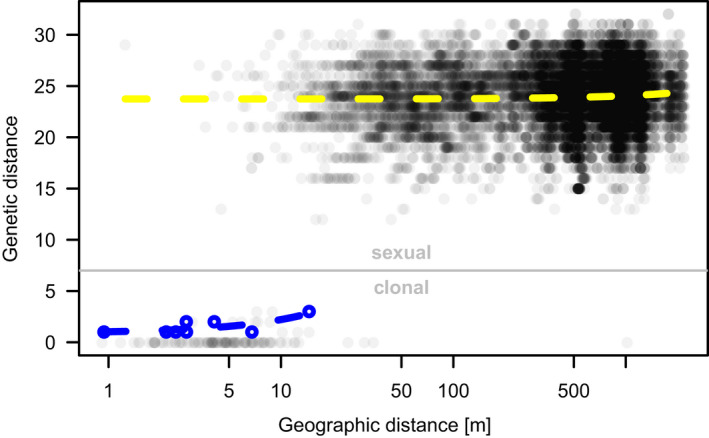
Pairwise genetic distances increase slightly with geographic distance for sexual reproduction pathways (yellow dashed line, upper part) but are more pronounced in groups with somatic mutations (blue dashed line, lower part). For the linear regression model of somatic mutations against geographic distance (blue points and dashed regression line), only the maximum values within each group of at least one allele difference were used

In general, allele differences accumulate with increasing geographic distance between potential sexually produced individuals (*F_df_*_=18,597_ = 44.29, *p* < .001, *R* ^2^ = .002376; Figure 8). Although the genetic distance did not clearly change with geographic distance when considering all clonal groups, it did significantly increase when considering only those individuals bearing somatic mutations (*N* = 8, *F_df_*_=6_ = 8.421, *p* = .02727, *R* ^2^ = .5839; Figures 7 and 8).

Pairwise genetic distances are small (*F*_ST_ < 0.03). The differentiation was least between the populations TY02‐VI and TY02‐VII compared to the larger region TY02 (*F*_ST_ ≈ 0.01).

## 4 DISCUSSION

### 4.1 Clonal growth is the dominant recruitment type and helps survival of refugial populations beyond the treeline

Based on our genotyping approach, we found evidence for sexual reproduction and dispersal in the focus region and increasing genetic difference among individuals with geographic distance. The results suggest that a majority of individuals in the study area belong to genetically identical groups although they were visually growing separately. The nuclear microsatellites applied in this study have been shown to be highly polymorphic for larch in this region (see also Kruse et al., 2018) and could differentiate the genotype of all individuals. Furthermore, they enabled the detection of clonal reproduction in our study and in a few cases mutations at one locus or more. The fragment length pattern indicates that these mutations are somatic and accumulated during lateral growth along their branches. Knowing that larch (*Larix* Mill.) can produce adventitious roots from branches (Cooper, 1911), it is thus very likely that we observe over very short distances of a few meters actively migrating krummholz individuals that reproduce by asexual cloning.

We find that most clones grow close to each other, within 15 m, which makes it likely that they actively grew over these distances. A few longer connections over tens of meters to, in one case, ~1 km are also seen. A likely explanation for the long‐distance separation is that reindeer damaged the plant and one part was transported, perhaps in the reindeer's antlers, and rerooted when it was dislodged. We saw many signs of damage to upright branches of krummholz that look like deer rubbing or gnawing (Figure 2b), so we think that reindeer activity (Kolpaschikov, Makhailov, & Russell, 2015; Pavlov, Kolpashchikov, & Zyryanov, 1996) could easily be a cause of the separation and rare long‐distance transportation of plant clones (cf. Cousens, Hill, French, & Bishop, 2010). Although the chance that a separated ramet can survive the translocation appears to be very low, it could explain the few observed long‐distance transportations, as the chance of misidentifying a clonal individual is extremely low. With this in mind, some ages might be overestimated, but based on the spatial configuration of clonal individuals the majority of spreading seems to stem from lateral growth (Figure 3).

The clonal growth mode has several advantages. Individuals lower their risk of extinction by having multiple occurrences of the same genotype in the landscape (Cooper, 1911). After roots have developed from a branch settled on the ground, new shoots will be connected to the original plant in the early stages. This would allow sharing of resources throughout the plant, so that under unfavorable microsite conditions both parent and clone could survive longer (Holtmeier & Broll, 2017; Öberg & Kullman, 2011). This plastic response makes sense in our study area, as harsh environmental conditions prevailed since the climate cooled at ~2,500 yrs BP (Klemm, Herzschuh, & Pestryakova, 2016). Our results suggest that this environmental change may have forced a selection for phenotypes (and with these genotypes) which were plastic in their growth, allowing new stems to grow if the main stem died during cold spells/extremes.

It is possible to misidentify sexually derived individuals as clones (Arnaud‐Haond, Duarte, Alberto, & Serrão, 2007). Although, during sexual reproduction, the offspring receives a genotype consisting of half the information from its mother and the other half from the father plant (Ashley, 2010; Selkoe & Toonen, 2006), there is an extremely rare chance that the resulting genotype is the same as one of the parents. This becomes more likely with low population numbers and thus higher chances of inbreeding. In this case, we would have observed intermediate genotypes with increasing numbers from identical/clonal to completely different/sexually produced individuals. This stands in contrast to our results, as we exhaustively sampled all living individuals present in the landscape and found only a few cases with allele differences at a maximum of three alleles, a pronounced gap of intermediate forms with 4 to 11 differences and all within a close distance (<14.5 m), so that a misidentification appears highly unlikely.

In contrast, three variants of asexual reproduction can occur and they become more common under stressful situations (Hartl & Clark, 2007). (A) Selfing is when a plant inseminates its ovules with its own pollen, although the likelihood of the offspring having an identical genetic identity is very low due to stochastic inheritance but increases in populations with a pronounced homozygosity. (B) Parthenogenesis is when a plant's noninseminated ovules develop into individuals either with a haploid or diploid chromosome set. (C) Clonal growth by producing stems other than the main branch from roots or from branches that have rooted themselves (Cooper, 1911), which, should the clones become disassociated, can give rise to genetically identical separate individuals. It is very unlikely that reproduction purely by selfing would yield the high rate of >50% clonal groups. Furthermore, the genetically identical individuals identified by microsatellite fragment lengths share the same diploid genotypes at all 16 loci and are mostly heterozygotic with a few somatic mutations. This suggests reproduction via parthenogenesis is highly unlikely.

In summary, plants beyond the treeline could act as nuclei for tundra colonization under current warming conditions, and consequently, the active spreading of clonal individuals that stabilize the refugial populations during colder phases might enable the treeline to advance quickly (as in Kharuk et al., 2006; Kruse et al., 2019). Alternatively, their adaptation to different/colder climates might not be easily overcome, and thus, they may slow down the treeline response to the current anthropogenic global warming.

### 4.2 Krummholz clonal groups conserve genotypes potentially over thousands of years

Our age determinations of branch sections gave a mean growth rate of 1.74 cm/yr. We used this lateral growth rate in our calculations as a first approximation, but the rate is likely to have been lower due to colder periods over the past ~2,500 years, which only ended with the strong warming of recent decades (Klemm et al., 2016). Variable growth rates will cause uncertainties in our age estimation, and we may have underestimated the age. Even a direct age determination of the oldest parts of an individual by radiocarbon dating may not resolve the true age of a plant as the oldest parts may have rotted (Öberg & Kullman, 2011). Another way of overestimating the age is when an individual that produced two clones that spread in opposite directions died and left no remains so that the full distance between the clones is used for age determination rather than the actual midway distance. In consequence, there is some doubt about whether all individuals originated from clonal growth after the pronounced environmental change in the centuries after ~2,500 years BP, although it is more likely as most estimated ages were <2,200 years.

Larches in Siberia can reach a very old age of up to 609 years when growing as upright trees (Vaganov et al., 1999 cited in Abaimov, 2010) and up to a thousand years for North American species (*Larix lyalii*, Arno & Habeck, 1972). Other individuals from the Pinaceae family attain ages of thousands of years (Rocky Mountain Tree Ring Research: Database of ancient trees (http://www.rmtrr.org/oldlist.htm, 2020) and when growing clonally (*Picea mariana*, 1,800 years, Laberge et al., 2000; *Picea abies*, 9,550 years, Öberg & Kullman, 2011).

In summary, the observed clonal individuals have survived over hundreds and potentially thousands of years. Throughout, they have conserved their genotype and they are now in a position to potentially recolonize the tundra under warming conditions. However, it remains an open question as to whether their cold environment adaptations will assist or restrict a tundra invasion. So far, the response to current warming of these krummholz sites has been hampered by seed limitations, but this may change in the coming decades (Brown et al., 2019; Wieczorek et al., 2017).

### 4.3 Implications of predominant asexual, clonal recruitment over the past millennia for current and future treeline responses

From its maximum extent 8,000 years ago, the northern Siberian treeline retreated about 200 km to its current position (MacDonald et al., 2008). The rapid temperature drop ~2,500 years BP (Klemm et al., 2016) seems to have caused a strong and fast larch population decline that coincided with the selection of suitable pheno‐/genotypes. This appears to have averted the expected heterozygosity depression observed in refugial populations with low population sizes due to inbreeding, as in our refugia we find very high genetic diversity (see also Kruse et al., 2018). Aside from the clonal individuals, we do not find a genetic imprint of asexual reproduction by the index of association (cf. Goss et al., 2014) and observe still a high level of heterozygosity under a high rate of clonal reproduction (cf. Balloux et al., 2003). Our findings suggest that asexual, clonal spread by layering was the preferred and predominant mode during the past two millennia, enabling individuals to survive for thousands of years. This led to the observed refugial populations far beyond the treeline in northern Siberia.

Recent climate warming has ameliorated the environmental conditions so that a northward migration of the treeline is likely to occur and could potentially expand beyond 200 km. Because refugia beyond the treeline can act as nuclei for tundra colonization (e.g., Holtmeier & Broll, 2005), the response speed can be much faster when environmental conditions improve than without refugia. However, field observations and modeling studies have shown that the treeline in northcentral Siberia is only migrating at a very slow rate of a few meters per year (Kharuk et al., 2006; Kruse et al., 2019), contrary to expectations.

**FIGURE 9:**
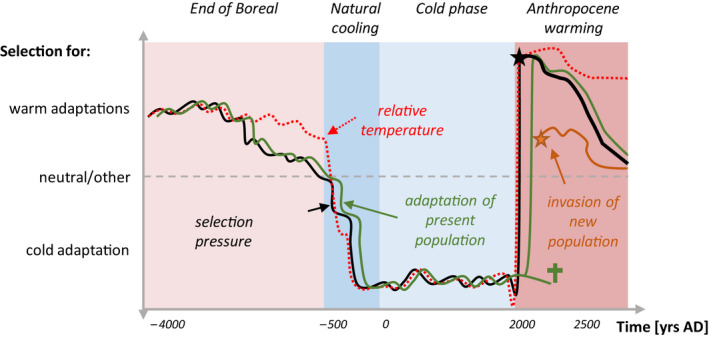
Conceptual diagram of selection pressure and corresponding adaptation in a population. The adaptation generally lags behind the selection pressure and decouples when constant mild conditions prevail. The current warming (black star) might be too rapid for the present locally adapted genotypes so they could go extinct (green cross). They may also hinder a faster tundra colonization of approaching genotypes via gene swamping. Alternatively, invasions of better‐adapted genotypes replace the local, now maladapted, population (orange star)

The colonization rate may speed up after a decadal‐long time lag if the populations can adapt and produce sufficient seed to increase in population number (Kruse et al., 2016, 2019; Wieczorek et al., 2017). This process may, however, be constrained by genetic maladaptation in the refugia. If their current survival is due to selection processes that have forced a plastic/phenotypic response, then the adaptations are reversible. Subsequently, with a change in environmental conditions, they can easily recolonize the tundra. In contrast, mutations that have allowed the genotype to survive can become fixed and inherited by the offspring (Figure 9). This genetic fixation might be further consolidated by sexual reproduction between relatives—which is quite likely in refugial populations due to smaller population sizes—and by the speed of environmental change causing severe diversity reductions (Arenas et al., 2012; Davis et al., 2015; Hartl & Clark, 2007). Such an adaptation of the genotype would result in a pronounced time lag between the change in environmental conditions and the population's ability to respond. The historically adapted populations may go extinct if the change is too rapid. The more likely response lies somewhere between these two extremes. The local, cold‐adapted genotypes may mix with approaching warm‐adapted genotypes, forming intermediate‐adapted genotypes—a process called gene swamping. This would still cause a slower treeline response than model predictions as they assume well‐adapted genotypes.

In conclusion, our understanding of the impact of millennia‐old genotypes in refugia beyond the treeline in northern Siberia, potentially maladapted to climate warming, is still too limited to give a clear estimate of the future migration response and fate of the refugia. It remains uncertain whether the refugial krummholz populations host reversible plastic adaptations to the cold phase and thus can still act as nuclei for rapid tundra colonization after a time lag needed for adjustment to novel warm conditions, or whether the adaptations are genetically fixed. Gene swamping, caused by mixing of the cold‐adapted genotypes with novel approaching genotypes, will delay treeline advance as more time is needed for the sexually reproduced individuals to spread through the landscape.

## CONFLICTS OF INTEREST

The authors declare no conflict of interest.

## AUTHOR CONTRIBUTIONS

**Stefan Kruse:** Conceptualization (equal); Data curation (lead); Formal analysis (lead); Funding acquisition (supporting); Investigation (equal); Methodology (lead); Project administration (equal); Validation (lead); Visualization (lead); Writing‐original draft (lead); Writing‐review & editing (equal). **Aleksey I. Kolmogorov:** Conceptualization (supporting); Investigation (equal); Writing‐review & editing (equal). **Luidmila A. Pestryakova:** Data curation (supporting); Funding acquisition (supporting); Investigation (supporting); Project administration (equal); Writing‐review & editing (equal). **Ulrike Herzschuh:** Conceptualization (equal); Formal analysis (supporting); Funding acquisition (lead); Investigation (equal); Methodology (supporting); Project administration (equal); Validation (supporting); Visualization (supporting); Writing‐original draft (supporting); Writing‐review & editing (equal).

## Supporting information

Appendix S1‐S5

## Data Availability

Sampling locations, morphological data, and microsatellite genotypes are stored publicly available on PANGAEA (https://doi.org/10.1594/PANGAEA.920680). The tree ring counts and positions of branch sections are stored on PANGAEA (https://doi.org/10.1594/PANGAEA.920838).
