# Supplementary material for: Long‐lived larch clones may conserve adaptations that could restrict treeline migration in northern Siberia
Source: Ecol Evol. 2020 Aug 17;10(18):10017–30. doi: 10.1002/ece3.6660 (PMC7520212; doi:10.1002/ece3.6660)
Supplement: Supplementary file 1 — Appendix S1‐S5 [file ECE3-10-10017-s001.docx]

# Supplement to Long-lived larch clones may conserve adaptations that could restrict treeline migration in northern Siberia

Running title: **Clonal growth in refugia impacts treeline migration**

**Stefan Kruse^1*^, Aleksey I. Kolmogorov^2^, Luidmila A. Pestryakova^2^, Ulrike Herzschuh^1,3,4^**

^1^ Alfred-Wegener-Institute, Helmholtz Centre for Polar and Marine Research

^2^ Institute of Natural Sciences, North-Eastern Federal University of Yakutsk, 677000 Yakutsk, Russia

^3^ Institute of Environmental Sciences and Geography, University of Potsdam, 14476 Potsdam, Germany

^4^Institute of Biology and Biochemistry, University of Potsdam, 14476 Potsdam, Germany

*****Correspondence: stefan.kruse@awi.de


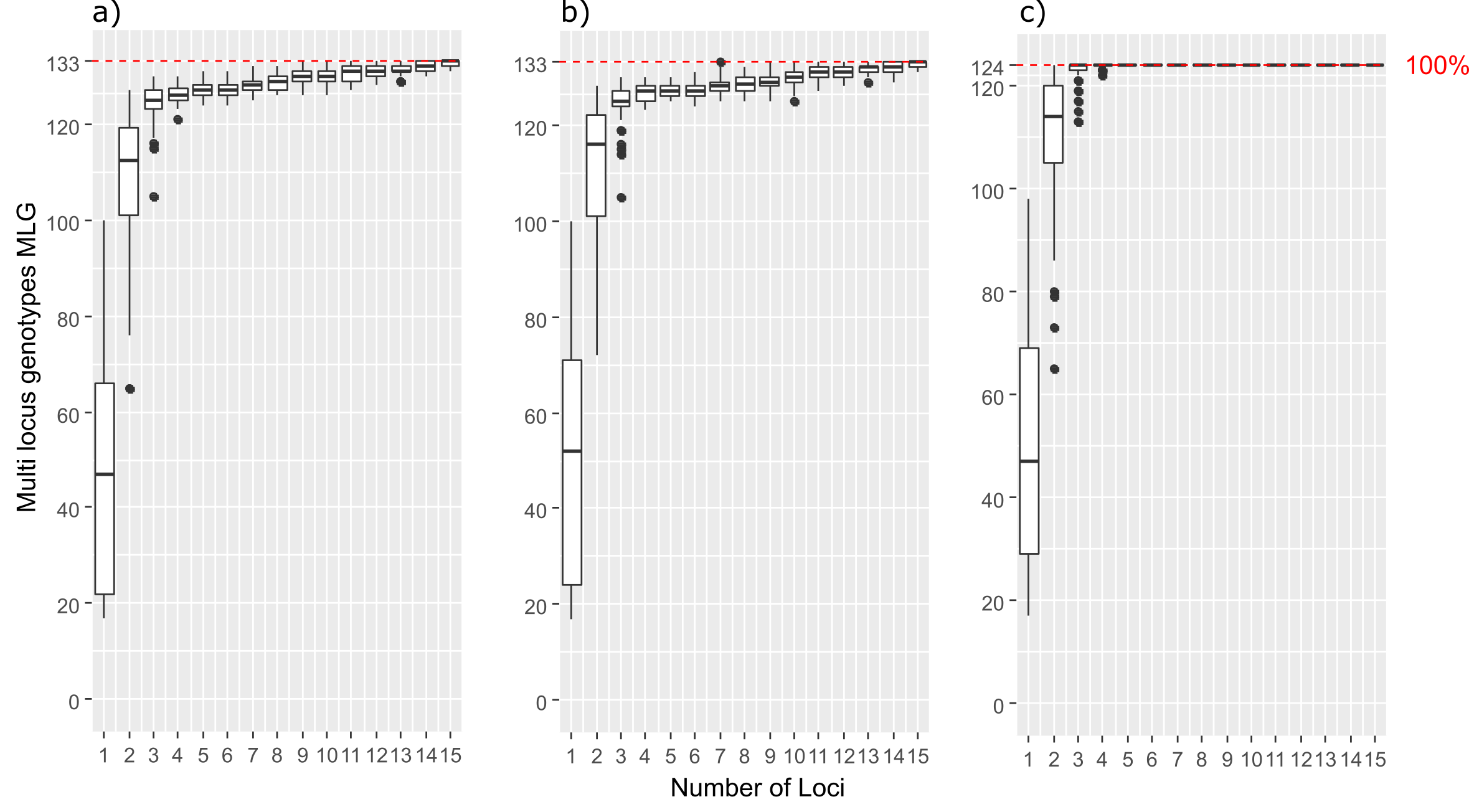


**Appendix 1. Genotype accumulation curve for the sample sets: a) all samples, b) clone-censored, and c) clone-censored and somatic mutation-censored.** Plateau reached at 16 loci.


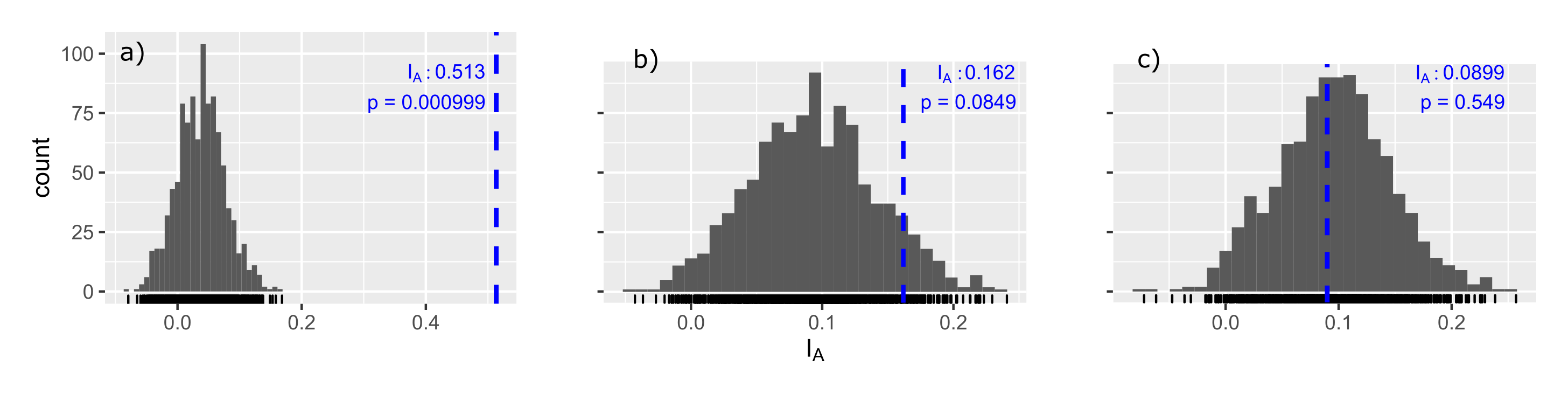


**Appendix 2. Index of association for three different sample data sets, a) all samples (N=194), b) clone-censored (N=137), and c) clone-censored and somatic mutation-censored (N=124).**


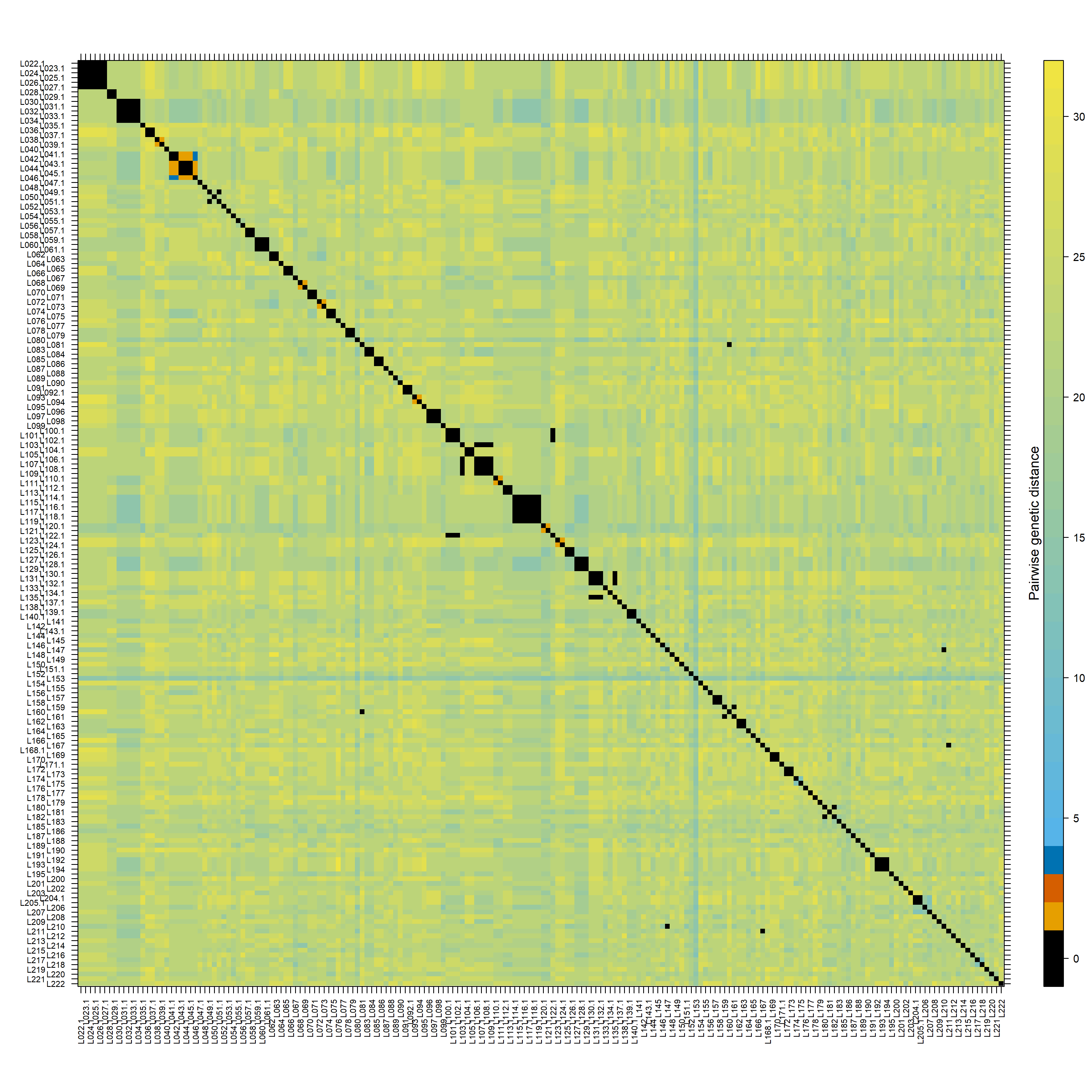


Appendix 3. Pairwise genetic distances across all unique individuals reveals clonal groups of identical (black) or small genetic differences (orange to dark blue).

See details in caption of Figure 4.

Appendix 4. Summary of pairwise genetic distance within clonal groups. In groups with more than two samples the pairwise distance can vary if somatic mutations occur in one or more individuals leading to different levels of genetic distances.

| Group name | Number of samples | Maximum allele difference (max:32) | Maximum genetic distance [%] | Levels of genetic distance |
| --- | --- | --- | --- | --- |
| L025 | 3 | 0 | 0 | 1 |
| L027 | 3 | 0 | 0 | 1 |
| L029 | 2 | 0 | 0 | 1 |
| L033 | 3 | 0 | 0 | 1 |
| L048 | 2 | 0 | 0 | 1 |
| L050 | 2 | 0 | 0 | 1 |
| L051 | 2 | 0 | 0 | 1 |
| L056 | 2 | 0 | 0 | 1 |
| L092 | 2 | 1 | 0.031 | 2 |
| L102 | 2 | 0 | 0 | 1 |
| L108 | 2 | 0 | 0 | 1 |
| L124 | 2 | 1 | 0.031 | 2 |
| L134 | 2 | 0 | 0 | 1 |
| L137 | 3 | 0 | 0 | 1 |
| L138 | 3 | 1 | 0.031 | 2 |
| L139 | 10 | 1 | 0.031 | 2 |
| L143 | 2 | 0 | 0 | 1 |
| L151 | 2 | 2 | 0.063 | 2 |
| L168 | 2 | 0 | 0 | 1 |
| L171 | 2 | 0 | 0 | 1 |


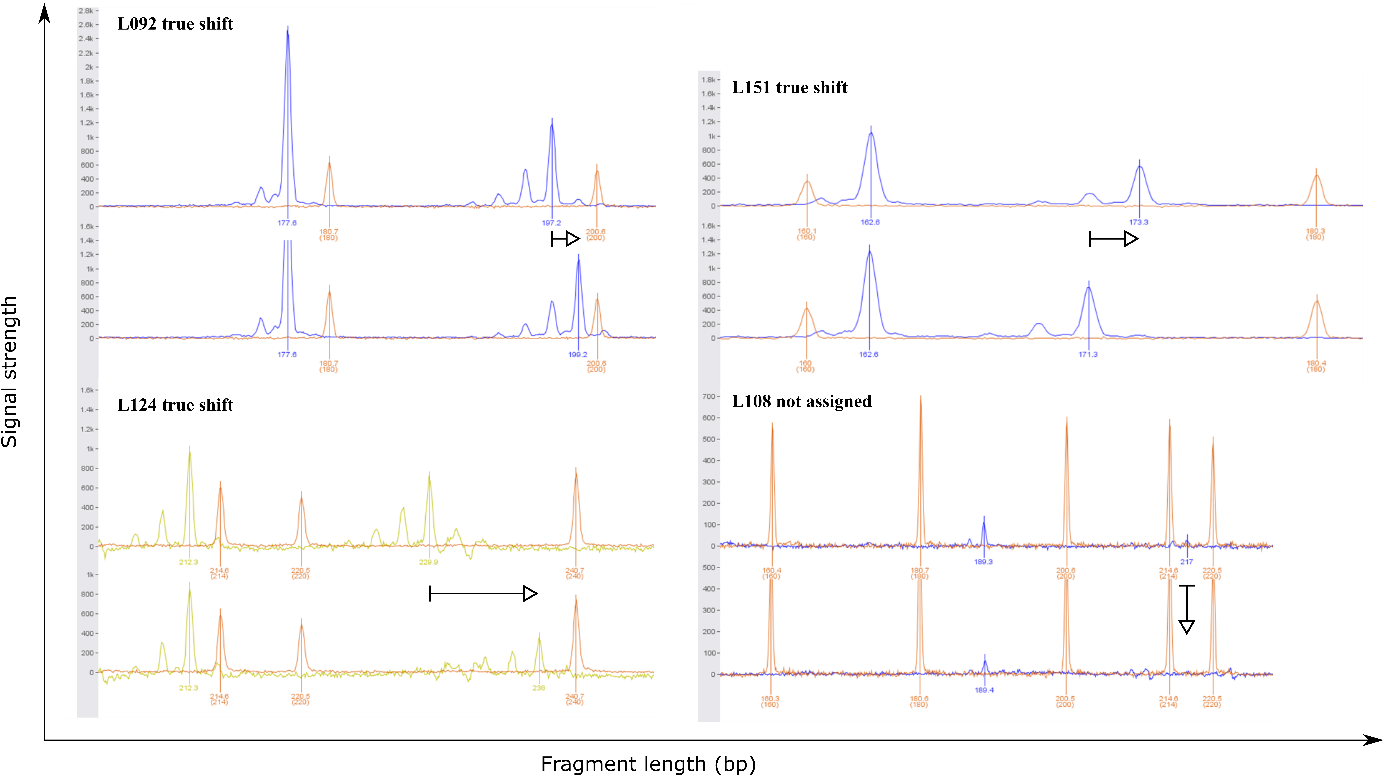


Appendix 5. Examples of microsatellite fragment length differences within clonal individuals.

Orange lines, size standard; blue and yellow lines microsatellites.
